# Brain Mechanisms of Altered Consciousness in Generalised Seizures

**DOI:** 10.3233/BEN-2011-0317

**Published:** 2011-03-29

**Authors:** Stefano Seri, Daniela Brazzo, Ngoc J. Thai, Antonella Cerquiglini

**Affiliations:** ^1^School of Life and Health SciencesAston Brain CentreAston Universityand Clinical Neurophysiology Unit BirminghamUK; ^2^Developmental Neurosciences UnitDepartment of Medical and Surgical BiotechnologySapienza UniversityPolo PontinoItaly

**Keywords:** Consciousness, generalized epilepsy, functional imaging

## Abstract

In spite of the inherent difficulties in achieving a biologically meaningful definition of consciousness, recent neurophysiological studies are starting to provide some insight in fundamental mechanisms associated with impaired consciousness in neurological disorders. Generalised seizures are associated with disruption of the default state network, a functional network of discrete brain areas, which include the fronto-parietal cortices. Subcortical contribution through activation of thalamocortical structures, as well as striate nuclei are also crucial to produce impaired consciousness in generalised seizures.

## 1. Introduction

The assessment of consciousness and the identification of its disturbances are key to a semiological distinction between epileptic seizure types. When differentiating simple from complex partial seizures, the ILAE Commission on Classification and Terminology in 1981 recognised the importance of an agreed and scientifically sound operational definition of consciousness. The differentiation between these two seizure categories was exclusively based on whether consciousness was preserved as opposed to absent or altered. To answer some of the criticisms on the vagueness of the proposed terminology, consciousness was defined in an appended glossary as the "degree of awareness and/or responsiveness of the patient to externally applied stimuli". Furthermore, responsiveness was specified as "the ability of the patient to carry out simple commands or willed movements", and awareness as "the patient's contact with events during the period in question". Even though these definitions were pragmatically helpful, they did not account for the complexity attached to the term consciousness [13]. The recent report of the ILAE commission tries to overcome this impasse by proposing a shift of focus from a distinction based on evaluation of consciousness to one in which differences in neural substrates are emphasised. Generalised seizures are therefore defined as "originating at some point within, and rapidly engaging, bilaterally distributed networks" [2].

In clinical practice consciousness is generally associated with the waking state and the ability to perceive, interact and communicate with the environment. It is quantified using the Glasgow Coma Scale, although measurement tools specific for epilepsy have recently been proposed [6].

From the pioneering studies of Moruzzi [28], which led to the definition of ascending reticular activating system (ARAS) as a central core in controlling the state of consciousness, we now have a clearer as well as more complex picture of the structure anatomically and physiologically involved in maintaining consciousness. Levels of consciousness are dependent on the integrity of brain systems and connections such as ascend-

ISSN 0953-4180/11/$27.50 © 2011 – IOS Press and the authors. All rights reserved ing brainstem/basal forebrain/thalamic pathways [31], widespread thalamocortical projections [7,27] as well as fronto-parietal cortical networks [17]. Intrinsic oscillatory properties of thalamic neurons have been suggested to govern their functional dynamics and regulate sleep and vigilance [20,32].

At the neural level, consciousness requires access to explicit representation of the sensory experience in parallel, distributed neural networks. To make matters more complex, the brain is capable of highly skilled, automatic, stereotyped and fluid behaviours without a conscious experience. From playing highly skilled sports to driving home along the same route every day, we can perform complex sensorimotor tasks without direct conscious access. Researchers refer to these behaviours as subserved by "zombie systems" [8] and its existence in the visual domain has been indirectly demonstrated [10]. It has been hypothesised that human behaviour is the result of an optimisation process between the actions of rapidly-responding stereotyped systems and intrinsically slower networks, which support thought processes and planning complex behaviour.

## 2. Cortical networks involved in the pathogenesis of generalised seizures

### 2.1. Absences

Absence seizures consist of brief episodes of staring, reduction of awareness and responsiveness, sometimes associated with eyelid fluttering or myoclonus, which can last 5 to 30 seconds. The altered consciousness may be isolated, such as in "simple" typical absences or it may be associated with clonic, tonic or automatic motor components such as in "complex" typical absences. The absence, whether simple or complex, is associated with widespread spike and wave EEG discharges at a regular rhythm of 3/second. Both human and animal studies have suggested that absence seizures are generated through abnormal network oscillations involving both the cortex and thalamus [9]. Attempts to define neural changes associated with the electro-clinical manifestation of absences seizures using neuroimaging techniques have generated controversial data. Some studies have shown a global ictal increase in cerebral metabolism or blood flow [29,33,35], others have detected spatially limited changes [5,11]. Typical absence seizures can often show fluctuation in EEG patterns, with non-fixed focal abnormalities [19]. The involvement of discrete brain regions might account for the variable degree of impairment of consciousness during absences seizures. Loss of consciousness in absence seizure has been related to the focal involvement of the bilateral association cortex, frontal neocortex and related subcortical structures disrupting normal information processing in these brain regions [3]. More specifically, bilateral activation of thalamus, insula, mesial frontal cortex and cerebellum coupled with bilateral parietal, anterior frontal and posterior cingulate deactivation suggest that changes in consciousness reflect the interruption of the default mode network (DMN) [15, 30] and thalamocortical activation [14]. The report of retained ability to perform a continuous performance task during brief spike-wave discharges in the presence of the typical functional MRI pattern of DMN interruption and fronto-thalamic activation [26] suggests that some abilities might be preserved in some patients in a "zombie brain" like modality.

Confirmation of the *cortical focus theory* [25] applied to the generation of spike and waves in absences seizures has emerged from analysis of connectivity patterns of electromagnetic signals across brain regions. The theory postulates an initial lead in a circumscribed area of the peri-oral region of the somatosensory cortex from which the activity rapidly spreads over the remaining cortical areas. This initial event produces a cascade within the intact thalamocortical network, which transforms the spike into spike-wave activity. A recent magnetoencephalographic study has identified a sequence of events associated with absences which include an initial long-range desynchronisation, followed by increased local synchronization and increased long-range synchronization [1]. This finding challenges the concept of spike-wave discharges being a result of a sudden and generalised synchronous activation and suggests the absences require interplay of complex and spatially distributed networks.

### 2.2. Generalised tonic clonic seizure

Whilst several studies have attempted to understand neural correlates of impaired consciousness in absences seizures few studies have investigated generalised tonic clonic seizures due to objective difficulties in examining the nature and mechanisms underlying the ictal impairment of consciousness [34]. Generalised tonic clonic seizures – the most typical type of convulsive seizures- represent an heterogeneous group, occurring in a variety of clinical conditions and epileptic syndromes. They have been traditionally divided into primarily generalised, in which there is no obvious focal onset, and secondarily generalised, in which seizures begin in a focal brain region and later spread to neighbouring and connected areas. The core of the attack consists of a stereotyped series of motor and autonomic events, which are associated with sudden loss of consciousness. On the EEG, the tonic phase is characterised by rhythmic 10 Hz activity that increases rapidly in amplitude and becomes discontinuous towards the end of the of the tonic phase (epileptic recruiting rhythm) [12], resulting in a reduction in temporal and spatial complexity of the brain electrical signal, necessary for maintaining consciousness [21]. During the clonic phase, bursts of 10 Hz rhythms become separated by intervals of inactive tracing or by slow waves. The bursts are synchronous with the jerks. At the end of a seizure, single spikes are common, and they may be followed by a slow wave, forming atypical spike wave formations. The convulsive event is preceded by a cascade of changes in sub-cortical excitability [18]. During the widespread and synchronous activation of the brainstem the thalamus and the cortex undergo a suppressed inhibitory state through the cholinergic activation, precipitating seizure discharge and possibly contributing to the loss of consciousness. Studies based on electrophysiological measures blood flow, and metabolic mapping suggest that the entire brain may be homogeneously involved in generalised tonic-clonic seizures [16,22,23], whereas animal data suggest more spatially confined regional changes [24]. A recent cerebral blood flow study in patients with secondary generalised seizures attempted to characterise neural changes with sufficient temporal resolutions to allow some inference on the specifics of the generalised phase. The study identified abnormally increased activity in subcortical structures such as cerebellum, basal ganglia, brainstem and thalamus, and again decreased activity in the association cortex associated with disruption of DMN [4].

## 3. Final comments

In spite of the relative paucity of studies on generalised seizures, particularly of convulsive type, important and converging data support the notion that generalised seizures, at the neural level, are associated with a profound and reproducible change in resting state activity, associated with a multifocal involvement of cortical and sub-cortical structures. These findings somewhat challenge the view of *centrencephalic epilepsy*, as well as posing some pressing questions on the biological validity of the classic and clinically useful dichotomy between focal and generalised epilepsies. These two clinical entities might share many elements in their biological substrate with clinical manifestations being the result of different timing and geometry of the involved networks.
